# Dynamic Functional Connectivity of Amygdala and Hippocampus in Posttraumatic Stress Disorder: A Multi-Site Study of the ENIGMA PTSD Consortium

**DOI:** 10.21203/rs.3.rs-10704000/v1

**Published:** 2026-09-03

**Authors:** Delin Sun, ENIGMA PTSD Working Group

**Affiliations:** Department of Psychiatry, Duke University School of Medicine, Durham, NC 27705 USA

**Keywords:** PTSD, Resting-State fMRI, Dynamic Functional Connectivity, Amygdala, Hippocampus

## Abstract

The amygdala and hippocampus are central to neural models of posttraumatic stress disorder (PTSD), yet their dynamic functional interactions with distributed brain networks remain poorly characterized, particularly in large, diverse samples. We analyzed resting-state functional MRI data from the ENIGMA PTSD Working Group, comprising 2,721 participants from 32 international sites: 1,017 individuals with PTSD (38.6±15.2 years; 52.0% female) and 1,704 controls (38.4±17.1 years; 45.8% female; 78.6% trauma-exposed). After standardized preprocessing with *HALFpipe*, we computed dynamic functional connectivity (DFC) as the standard deviation of functional connectivity across sliding windows (window length = 40 seconds, overlap = 50%) for bilateral amygdala and hippocampal seeds across 1,726 seed-to-parcel connections spanning cortical, subcortical, and cerebellar regions. Linear mixed-effects models through *IBMMA* tested the effect of PTSD diagnosis across all connections, and within significant connections, secondary analyses examined modulation by sex, age, index trauma, depression severity, and childhood trauma severity, along with associations with overall PTSD symptom severity and symptom clusters (B–E). All findings survived false discovery rate correction (*q* < .05). Individuals with PTSD compared to controls showed greater DFC between the seeds and 22 regions spanning control, salience/ventral attention, dorsal attention, somatomotor, default mode, and visual networks. Case-control differences were moderated by sex and present in males only. PTSD severity and symptom clusters were positively associated with most of the connections. These findings indicate that PTSD is accompanied by greater temporal variability in connectivity between amygdala and hippocampal seeds and widespread brain networks.

## Introduction

Posttraumatic stress disorder (PTSD) is a severe mental health condition that develops following exposure to life-threatening events, serious injury, or sexual violence (1). PTSD causes significant functional impairment across family, occupational, and social domains (2), and is associated with elevated rates of suicide (3), interpersonal violence (4), and cardiometabolic morbidity (5). Consequently, substantial efforts have focused on identifying neurobiological markers for diagnosis and treatment development (6).

The amygdala and hippocampus, core limbic regions implicated in neural processing of emotion and memory, occupy central roles in neurocircuitry models of PTSD (7,8). These models posit that PTSD reflects a hyperactive amygdala that drives exaggerated fear and threat responses, together with deficient hippocampal function that impairs the contextual modulation of fear and the discrimination of safe from dangerous environments. Consistent with these roles, the amygdala mediates fear learning, threat detection, and defensive behaviors (8,9), while the hippocampus supports contextual processing of fear memories (10). Neuroimaging studies have consistently demonstrated PTSD-related alterations in these structures, including reduced volume (11–13), heightened amygdala reactivity to threat-related stimuli (14,15), and disrupted hippocampal engagement during fear conditioning (16). Importantly, these regional alterations do not occur in isolation but reflect broader disruptions in the functional interactions between the amygdala, hippocampus, and distributed brain networks.

Human cognition and emotion processing depend on precisely coordinated neural interactions across distributed brain networks (17). Resting-state functional connectivity, which quantifies temporal correlations between brain regions using functional magnetic resonance imaging (fMRI), offers a powerful approach for characterizing these interactions while avoiding confounds related to task performance and participant compliance (18). Traditional applications of this method focus on static functional connectivity (SFC), which assumes temporal stability and collapses inter-regional time-series correlations across the entire scanning session. Using the largest PTSD-related resting-state fMRI dataset assembled to date (N = 2,721) aggregated by the ENIGMA PTSD Working Group, Hinojosa et al., (19) recently reported greater SFC in PTSD patients between the left amygdala seed region and clusters of voxels in the right hippocampus, right amygdala as well as bilateral lingual gyri. That analysis, however, characterized only time-averaged (static) connectivity and did not address how these interactions fluctuate over time.

Dynamic functional connectivity (DFC) measures how functional connectivity fluctuates over the time course through approaches such as sliding time windows. This temporal variability in connectivity, measured by the standard deviation of connectivity across sliding time windows, has been linked to a range of cognitive and behavioral measures in healthy individuals, including sustained attention, processing speed, fluid intelligence, and cognitive flexibility (20,21). Greater DFC does not inherently map to superior or impaired performance, depending on the cognitive domain and disorder context, higher temporal dynamics can signal either suboptimal network coordination (22,23) or an adaptive mechanism that maintains functions (24,25). Importantly, DFC has been shown to differentiate participants with comorbid PTSD and mild traumatic brain injury from controls more sensitively than SFC in a large multisite sample (26), suggesting DFC may be a valuable tool for characterizing dynamic neural processes in PTSD. However, DFC studies in PTSD have yielded mixed results. Jin et al., (27) documented a pattern of weaker DFC alongside stronger SFC in PTSD relative to controls, though the group differences did not reach statistical significance. Fu et al., (28) reported region-specific alterations of mixed directionality, with PTSD patients showing DFC greater between the precuneus and insula but decreased between the precuneus and inferior parietal lobe. Dai et al., (29) reported DFC alterations concentrated within the default mode network (DMN) and between the DMN and cerebellar regions in PTSD.

These inconsistencies likely reflect methodological heterogeneity, varied sample characteristics, and limited sample sizes (30). Notably, few prior studies examined DFC of the amygdala or hippocampus specifically, a surprising gap given the central role of these two structures in PTSD. To address these gaps, utilizing the largest PTSD-related resting-state fMRI dataset (N = 2,721) that underwent standardized preprocessing and rigorous quality control (19), we investigated PTSD diagnosis and symptom severity associations with DFC for bilateral amygdala and bilateral hippocampus seeds with cortical, subcortical, and cerebellar regions. Following established neurocircuitry models of PTSD (9,31), which emphasize dysregulated communication between prefrontal regions and subcortical threat- and memory-related hubs, we hypothesized that individuals with PTSD show greater DFC between prefrontal cortex and both the amygdala and hippocampus seeds, reflecting greater temporal variability in the coupling of these systems at rest. We further explored potential moderating effects of sex, age, index trauma, depression severity, and childhood trauma exposure on PTSD-related DFC, and whether DFC scaled with symptom severity.

## Methods

### Participants

Clinical and neuroimaging data were aggregated by the ENIGMA-PGC PTSD Working Group from 32 international study sites (Fig. 1A). The final sample (**Table 1**) included 1,017 individuals with PTSD (52.0% female) and 1,704 controls (45.8% female; 78.6% trauma-exposed). Participants were assigned to the PTSD group based on meeting full diagnostic criteria using site-specific assessment instruments. Site-specific inclusion/exclusion criteria, sample characteristics and clinical assessments, and scanning parameters are summarized in **Supplementary Tables S1–S4**, respectively. All data were collected under approval from local institutional review boards or ethics committees, and written informed consent was obtained from all participants. Secondary analysis of the aggregated dataset was granted exempt status by the Duke University Health System IRB.

### Harmonization of Multisite Clinical Data

All symptom severity scores, drawn from multiple sites using diverse instruments with different score ranges, were linearly normalized to a 0–1 scale using [S − Min(S)] / [Max(S) − Min(S)], where S is the original score, and Min(S) and Max(S) are the minimum and maximum possible scores of the clinical instrument used.

PTSD symptom severity was assessed using the Clinician-Administered PTSD Scale (CAPS-IV or CAPS-5) (32,33) or the PTSD Checklist (PCL or PCL-5) based on DSM-IV or DSM-5 criteria (34,35). All CAPS-IV data were converted to CAPS-5 at the total and item levels using previously reported conversion methods (36–38). Scores for symptom clusters B, C, D, and E were calculated directly from DSM-5 item scores. Site-specific clinical instruments for PTSD diagnosis, symptom severity, depression, and childhood trauma, along with the number of participants assessed with each tool per site, are summarized in **Supplementary Table S2**.

### Neuroimaging Data Preprocessing

Resting-state whole-brain T2*-weighted generated Blood Oxygen Level Dependent (BOLD) images were acquired at each site and shared with the ENIGMA PTSD Working Group for centralized preprocessing. Multi-site fMRI data were preprocessed using ENIGMA *HALFpipe* (39), which leverages *fMRIPrep* (40) to ensure standardized and reproducible processing. Preprocessing included spatial smoothing with a 6 mm FWHM Gaussian kernel, grand mean scaling, registration to MNI152NLin2009cAsym standard space, temporal filtering with a Gaussian kernel (128 s FWHM) to remove low-frequency noise, and anatomical component-based noise correction (aCompCor).

Quality control procedures are illustrated in Fig. 1B. Participants were excluded based on the following criteria: pipeline completion errors (0.6% of all subjects), anatomical issues during skull stripping (2.0%), segmentation failures (2.8%), registration failures (2.6%), excessive head motion (more than 30% of frames with framewise displacement exceeding 0.5 mm; 4.5%), low temporal signal-to-noise ratio (tSNR outside the interquartile range; 1.0%), or excessive noise components (more than 85% of ICA components flagged; 8.4%). Quality control thresholds were determined empirically based on the distribution of the full dataset.

### Atlas-Based Dynamic Functional Connectivity

BOLD signal time courses were extracted from 400 cortical parcels defined by the Schaefer atlas (Schaefer et al., 2018), grouped according to Yeo’s 17-network solution (41); 17 subcortical regions from the FreeSurfer anatomical segmentation (Aseg) atlas (42), which include the four seed regions (left and right amygdala and hippocampus); and 17 cerebellar regions from the Buckner atlas (43); see **Supplementary Fig. S1**. The four seed regions, left and right amygdala and hippocampus, were derived from the Aseg atlas (42). We examined both seed-to-seed (n = 6) and seed-to-region connections (n = 4 × 430 = 1,720), yielding 1,726 connections in total.

Dynamic functional connectivity was estimated using a sliding time window approach, in which the full time course was segmented into overlapping time windows and Pearson’s correlation coefficients were computed within each window. DFC was then quantified as the standard deviation of these correlation coefficients across all windows. The main analysis used a window length of 40 seconds and 50% overlap between adjacent windows. This window length is sufficient to capture transient fluctuations in functional connectivity while yielding stable correlation estimates consistent with known neural dynamics (44,45). A 50% overlap balances temporal resolution with computational efficiency across sites with variable scan durations (46). Site-specific DFC parameters including repetition time (TR) and the number of sliding windows are summarized in **Supplementary Table S4**.

### Statistical Modelling

All statistical models are summarized in **Supplementary Table S5**.

#### Primary Analysis.

Linear mixed-effects models implemented in the *IBMMA* package (47) examined case-control differences in DFC, with age, age^2^, sex, and a diagnosis × sex interaction term as fixed effects, and study site as a random intercept. Age^2^ was included to capture nonlinear age effects. The diagnosis × sex interaction was included given well-established sex differences in PTSD neurobiology (48,49), which we reported previously using seed-to-voxel analysis of this dataset (19). False discovery rate (FDR) correction (*q* < .05, two-tailed) (50) was applied across all 1,726 connections.

#### Robustness of DFC Findings.

To assess robustness of DFC results to sliding window parameter selection, primary analyses were repeated across 12 parameter combinations (window lengths of 30, 40, 50, and 60 seconds × overlap of 25%, 50%, and 75% of the window length). For each connection, two complementary indices of robustness were computed: (1) the proportion of parameter combinations yielding FDR-corrected significance (*q* < .05), as a descriptive index of replicability across parameter space; and (2) the Stouffer-combined p-value, which is synthesized from the individual p-values corresponding to each of the 12 parameter combinations (51). Stouffer’s method uses results from multiple tests of the same null hypothesis to boost power by combining the joint probabilities offered from their converging evidence. The resulting combined p-values were then FDR-corrected across all tested connections to control for multiple comparisons. A DFC finding from the primary parameter set (40-second window, 50% overlap) was considered robust if it met all of the following criteria: *i)* FDR-corrected significance with primary parameters, *ii)* FDR-corrected significance in the Stouffer-combined analysis, *iii)* consistent direction of effects across all 12 combinations, and *iv)* a significance ratio of at least one-third (> 4 of 12 combinations).

#### Sensitivity Analysis.

Sensitivity analyses examined whether the primary PTSD diagnosis effects were robust to potential confounds, restricted to connections surviving FDR correction (*q* < .05) in the primary analysis. We conducted five analyses: (1) adding mean framewise displacement as a covariate to rule out motion artifacts; (2) adding TR as a covariate to account for differences in temporal resolution; (3) restricting to adults (age ≥ 18 years) to address developmental heterogeneity; (4) excluding controls (n = 21) with subthreshold PTSD to assess their influence on the observed effects; and (5) excluding trauma-unexposed participants (n = 365) to directly compare trauma-exposed individuals with versus without PTSD. FDR correction (*q* < .05) was applied separately within each analysis. No correction was applied across the five analyses, as they represent sensitivity checks of the same finding under alternative assumptions rather than independent hypothesis tests.

#### Moderation Analysis.

Moderation analyses examined whether PTSD diagnosis effects were moderated by sex, age, index trauma type (military versus civilian), depression severity, or childhood trauma severity. All moderation analyses were restricted to connections showing significant PTSD diagnosis effects and were conducted separately for each moderator. Sex moderation was evaluated via the diagnosis × sex interaction term to account for established sex differences in PTSD prevalence (19,52). For the remaining four moderators, a three-way interaction term (diagnosis × sex × moderator) was added to the primary model, with all lower-order terms retained, to assess whether each moderator influenced PTSD-related connectivity alterations and whether this relationship differed by sex.

FDR correction (*q* < .05) was applied separately for each moderator across all tested connections. To control for multiple comparisons across the five moderators, a threshold of *q* < .01 was applied (*i.e*., .05 divided by five moderators), maintaining an experiment-wide error rate of *q* < .05. For connections showing significant moderation effects, post-hoc analyses were conducted to characterize the direction and nature of the interaction. These post-hoc analyses were not subject to additional correction, as they were conducted solely to interpret significant effects rather than to test new hypotheses.

#### Dimensional Analysis.

To complement the categorical diagnosis approach, separate linear mixed-effects models examined dimensional associations between DFC and PTSD symptom severity. The primary diagnostic analysis addresses whether PTSD as a categorical entity is associated with DFC, while dimensional analysis addresses whether DFC varies linearly with symptom burden (53,54). These analyses address distinct scientific questions rather than serving as replications of one another. They also draw on partially non-overlapping samples, because symptom severity data were available for only a subset of participants (see **Table 1**), whereas the diagnostic analysis included all participants. Models were run across all participants with available severity data and included PTSD total symptom severity and each symptom cluster severity (Clusters B–E: intrusion, avoidance, negative alterations in cognition and mood, and hyperarousal/reactivity) as predictors of interest. The PTSD severity sex interaction term, age, age†, and study site as a random intercept were included in all models, consistent with the primary analysis. Separate models were run for each symptom cluster to avoid multicollinearity. FDR correction (*q* < .05) was applied across tested connections per symptom cluster. To further control for multiple comparisons across the five severity measures, a threshold of *q* < .01 was applied (i.e., .05 divided by five severity measures), maintaining an experiment-wide error rate of *q* < .05.

### Results Reporting

Results are reported as significant DFC associations for seed-to-parcel pairs. Each Schaefer parcel is identified by its Yeo 17-network designation and parcel name **(Supplementary Fig. S1A**); because many parcels carry only a network name with a numbered suffix (e.g., SomMotA-16) rather than a descriptive anatomical label, the overlapping AAL (Automated Anatomical Labeling) atlas region(s) are additionally provided for anatomical reference.

In addition to the DFC analysis, we computed static FC (SFC) between the same seed regions and atlas parcels using the identical parcellation and mega-analytic model, to provide a within-sample static reference (**Supplementary SFC findings**).

## Results

### Descriptive Statistics

Diagnostic groups were well-matched on age but differed in sex distribution, with a higher proportion of females in the PTSD group. Individuals with PTSD exhibited significantly higher severity scores across all clinical measures including PTSD total symptoms, all PTSD symptom clusters, depression, and childhood trauma (all *p*’s < .001). Importantly, there was no significant difference in framewise displacement between diagnostic groups during fMRI acquisition, indicating comparable head motion (**Table 1** and **supplementary Fig. S2**).

### Primary Analysis

Of all 1,726 DFC connections examined, 54.3% showed statistically robust group differences (Stouffer FDR-corrected *p* < .05), 67.6% showed consistent effect direction across all 12 parameter combinations, and 53.0% met both criteria simultaneously. Participants with PTSD demonstrated greater DFC in 22 connections that met all four robustness criteria (*t* ’s > 3.041, *q*’s < 0.049, Cohen’s *d* range: 0.121 – 0.195; Fig. 2 and **Table 2**).

#### Left Amygdala Seed.

PTSD was associated with greater DFC between the left amygdala and parcels spanning multiple networks (*t*’s > 3.130, *q*’s < .044, Cohen’s *d* range: 0.127–0.195), including control networks (left and right lateral prefrontal cortex in control network A; left ventrolateral prefrontal cortex and bilateral inferior parietal lobule in control network B), default mode network B (left ventral and dorsal prefrontal cortex), salience/ventral attention network A (left frontal opercular/insular region), somatomotor network A (right somatomotor cortex), dorsal attention network A (right superior parietal lobule), and central visual network (bilateral extrastriate cortex).

#### Right Amygdala Seed.

The PTSD group exhibited greater DFC between the right amygdala and somatomotor networks (left auditory cortex and right secondary somatosensory cortex in network B; left primary somatomotor cortex in network A) (*t*’s > 3.136, *q*’s < .044, Cohen’s *d* range: 0.127–0.166).

#### Left Hippocampus Seed.

PTSD was accompanied with greater DFC between the left hippocampus and the left inferior parietal lobule in control network B (*t* = 3.041, *q* = .049, Cohen’s *d* = 0.121).

#### Right Hippocampus Seed.

Individuals with PTSD showed larger DFC between the right hippocampus and salience/ventral attention networks (left parietal operculum and right posterior medial cortex in network A), somatomotor networks (left auditory cortex and right secondary somatosensory cortex in network B), default mode network B (left dorsal prefrontal cortex), and peripheral visual network (left superior extrastriate cortex) (*t*’s > 3.456, *q*’s < .027, Cohen’s *d* range: 0.137–0.190).

### Sensitivity Analyses

Case-control differences remained significant when (1) including mean framewise displacement as a covariate (*t*’s > 2.983, *q*’s < .003), (2) adding TR as a covariate (*t*’s > 2.362, *q*’s < .018), (3) restricting to adults only (*t*’s > 2.514, *q*’s < .012), (4) removing the subthreshold individuals (*t*’s > 3.042, *q*’s < .002), and (5) removing trauma-unexposed participants (*t*’s > 2.708, *q*’s < .007). Together, these results indicate that the primary findings were robust and were not attributable to head motion, scanning temporal resolution, developmental heterogeneity, subthreshold individuals, or trauma exposure status.

### Moderation Analyses

#### Sex Moderation.

Among 22 connections, 18 exhibited significant PTSD diagnosis × sex interaction (*t* ’s < −2.116, *q*’s < .042; **Table 3**). Post hoc analyses revealed stronger DFC in males with PTSD relative to male controls (*t* ’s > 2.964, *p*’s < .003), whereas no case-control difference was observed in females (|*t|*’s < 1.475, *p*’s > .141).

#### Age Moderation.

A significant DFC interaction was detected between the right amygdala and somatomotor networks (left auditory cortex in network B; *t*(2585) = −3.267, *q* = .024). However, post hoc examination revealed no significant age-related changes within either the control (*t*(1575) = 0.163, *q* = .871) or PTSD (*t*(907) = 0.103, *q* = .918) group separately.

#### Index Trauma, Depression, and Childhood Trauma Moderations.

No significant PTSD diagnosis × index trauma type interaction (*|t|*s < 2.942, *q*’s > 0.073), PTSD diagnosis × depression severity interaction (*|t|*s < 1.730, *q*’s > .708), or PTSD diagnosis × childhood trauma severity interaction (*|t|*s < 2.419, *q*’s > .347) was detected.

### Dimensional Analyses

As shown in Fig. 3, PTSD symptom severity was positively associated with 21 of 22 DFC connections (*t* ’s > 2.466, *q*’s < .014; **Table 3**) across participants.

At the symptom cluster level, cluster C (avoidance) showed the broadest association, reaching significance for all 22 connections (*t*’s > 2.864, *q*’s < .004). Cluster B (intrusion) and Cluster D (negative alterations in cognition and mood) severity were each associated with 18 of 22 connections (*t*’s > 2.047, *q*’s < .05 and *t*’s > 2.140, *q*’s < .04, respectively). Cluster E (hyperarousal) showed significant associations in 10 of 22 connections (*t*’s > 2.347, *q*’s < .042). Full results are presented in **Table 3**.

### SFC Findings

Atlas-based SFC analyses identified greater SFC between left and right amygdala seeds (**Supplementary SFC findings**), a substantially narrower pattern than the DFC results.

## Discussion

This large-scale mega-analysis examined DFC in the same resting-state fMRI dataset used by Hinojosa et al., (19) for their SFC analysis, aggregating data from 2,721 participants across 32 international sites within the ENIGMA PTSD Working Group. Participants with PTSD compared to controls showed greater DFC for the amygdala and hippocampus seeds across 22 connections (Cohen’s *d* range: .121–.195). These connections spanned both prefrontal and extra-prefrontal regions, distributing across the control, salience/ventral attention, dorsal attention, somatomotor, default mode, and visual networks. Analyses restricted to these 22 connections showed that sex moderated PTSD-related differences in 18 connections, with case-control differences present in males but not females. Dimensional analyses revealed that greater overall PTSD symptom severity was positively associated with 21 connections. The avoidance cluster (Cluster C) showed the most widespread positive associations with all 22 connections, followed by intrusion (Cluster B) and negative alterations in cognition and mood (Cluster D), each associated with 18 connections. The hyperarousal cluster (Cluster E) showed significant associations in 10 connections.

Individuals with PTSD demonstrated greater DFC across 22 connections spanning all four seed regions. This pattern was markedly broader than the static findings of Hinojosa et al. (19), who reported greater SFC in PTSD confined to the left amygdala seed and a small set of targets (right hippocampus, right amygdala, and bilateral lingual gyri). Our own atlas-based SFC analysis (**Supplementary SFC findings**) similarly detected greater SFC only between the left and right amygdala. Our *a priori* hypothesis was only partially supported. Prefrontal DFC alterations were concentrated in the left amygdala seed, with limited involvement of the right hippocampus, and were absent for the right amygdala and left hippocampus. Medial prefrontal regions central to prevailing neurocircuitry models of PTSD (9,31) did not reveal significant case-control differences in DFC, whereas the prefrontal effects we observed instead involved lateral and orbital frontal cortices, and the broader pattern extended well beyond prefrontal-limbic circuits. This dissociation is noteworthy given commentary that amygdala and hippocampus connectivity alterations in PTSD at rest may be circumscribed to medial temporal and occipital regions (55). The present findings challenge this view, showing that PTSD-related temporal variability in seed-based connectivity extends broadly across the control, salience/ventral attention, default mode, somatomotor, and visual networks, underscoring DFC as complementary to static functional connectivity. Notably, a data-driven investigation of whole-network dynamics in a partially overlapping ENIGMA-PGC sample found no robust case-control differences in dynamic graph metrics or recurrent connectivity states (56). This divergence suggests that DFC alterations in PTSD may be regionally specific to amygdala- and hippocampus-centered circuits rather than global network reorganization. Such focal effects may be difficult to detect in data-driven whole-network approaches, whether because they are genuinely diluted at the whole-connectome level or because the greater multiple-comparison burden of whole-network analyses reduces sensitivity to circumscribed effects. Convergence between diagnostic and dimensional analyses further strengthens confidence that our DFC alterations track clinical status rather than noise.

A striking feature of these results is their uniform direction, with all 22 connections showing greater DFC in PTSD and none showing the reverse. We caution against equating greater temporal variability with dysfunction. Greater variability may index instability in network coupling (22,23), but it may equally reflect a compensatory response preserving function despite disruption elsewhere in the circuit (24,25). Two design features limit resolution of this ambiguity. First, most controls are trauma-exposed (78.6%), and excluding trauma-unexposed controls did not alter the significance of the case-control differences; the reported effects therefore reflect PTSD versus trauma-exposed controls, so the difference may reside in the controls as readily as in the cases: lower DFC in controls could reflect successful adaptation, with the elevated variability in PTSD representing an unresolved state, though the converse is equally admissible. Second, the cross-sectional design cannot establish whether these alterations precede trauma exposure or follow PTSD onset. Methodological explanations are unlikely to account for the unidirectional pattern, as head movement reflected by framewise displacement did not differ between groups, effects were robust to framewise displacement as a covariate, and findings held across 12 sliding-window parameter combinations. We therefore frame the interpretations below as hypotheses about the systems involved rather than as evidence of pathology. The annotations map each seed’s DFC alterations onto the functional systems in which they fall, describing where temporal variability is elevated rather than advancing a mechanism for each connection, and the cited studies situate rather than confirm these patterns.

### Left Amygdala.

Greater DFC of the left amygdala with inferior frontal, orbitofrontal, and superior frontal cortices (default mode network B and control network B) falls within systems associated with emotion regulation, value-based processing, and cognitive control (9). Elevated temporal variability in amygdala coupling with inferior parietal and angular regions (control networks A and B) may further reflect variability in attentional control and contextual integration circuits, consistent with PTSD-related attention network dysregulation (57).

Greater DFC of the left amygdala with bilateral extrastriate cortex (central visual network) extends prior evidence of altered amygdala-visual functional connectivity (19), and demonstrates that the temporal dynamics of this coupling are also altered in PTSD. Our finding comports with evidence that early visual cortex structural integrity is negatively associated with PTSD severity (58) and that fear generalization engages ventral visual stream circuitry (Morey, et al., 2020). Greater DFC with the left inferior frontal operculum adjacent to the insula (salience/ventral attention network A), right superior parietal lobule (dorsal attention network A), and bilateral premotor and somatosensory cortex (control network A and somatomotor network A) may reflect greater temporal variability in coupling with systems mediating interoceptive awareness, salience detection, and somatic arousal. Our finding is consistent with the insula’s role as a convergence hub for these functions (60), and consistent with evidence that traumatic memories in PTSD are characterized by dysregulation of sensorimotor and interoceptive processes (61).

### Right Amygdala.

In contrast to the broad cortical distribution of left amygdala DFC effects, greater DFC of the right amygdala was confined to somatomotor and auditory networks. This restricted distribution is consistent with the proposed specialization of the right amygdala in sensory-mediated fear expression and reflexive autonomic responses to threat (62), and aligns with evidence of altered somatomotor network connectivity and heightened subcortical sensory responsivity in PTSD (15). Greater temporal variability in right amygdala coupling with auditory regions, including left rolandic operculum and superior temporal cortex and right primary auditory cortex (somatomotor network B), and with left premotor cortex (somatomotor network A), may contribute to aberrant sensory processing and threat-related motor readiness in PTSD (9). Positive associations with total symptom severity and with all four symptom clusters suggest these alterations scale with clinical symptoms. Given the right amygdala’s role in sensory fear circuitry, these lateralized connectivity patterns may have therapeutic relevance. Notably, personalized fMRI-guided TMS targeting the right amygdala threat circuitry has demonstrated reductions in amygdala reactivity and long-term PTSD symptom improvement (63).

### Left Hippocampus.

Left hippocampus DFC was limited to a single connection with the left inferior parietal lobule (control network B), which was positively associated only with the avoidance cluster. This restricted distribution is consistent with the functional specialization of the left hippocampus in verbal, sequential, and language-related memory processing (64,65). The inferior parietal lobule supports working memory, attentional control, and contextual integration, functions that vary with PTSD symptom severity (57). Greater temporal variability in this coupling with avoidance severity may reflect less stability in hippocampally-mediated contextual processing, potentially related to impaired discrimination of safe from threatening contexts and the consequent behavioral avoidance that relates to PTSD.

### Right Hippocampus.

The distribution of the right hippocampus DFC was broader than the left hippocampus, spanning salience/ventral attention, somatomotor, default mode, and peripheral visual networks. It is consistent with the established role of the right hippocampus in spatial memory, scene construction, and anxious arousal under high-anxiety conditions (65). Higher temporal variability in coupling with left superior frontal gyrus (default mode network B) may reflect altered hippocampal-prefrontal coordination supporting episodic memory retrieval and suppressing intrusion symptoms. Furthermore, it is consistent with evidence that resting-state hippocampal-prefrontal coupling relates to the emotional intensity and sensory-perceptual properties of intrusive trauma memories in PTSD (66,67). Greater DFC with the bilateral rolandic operculum and superior temporal cortex (somatomotor network B), right supplementary motor area (salience/ventral attention network A), and left supramarginal gyrus (salience/ventral attention network A) suggests broader involvement of sensorimotor, motor planning, and auditory-parietal circuits, paralleling the right amygdala-somatomotor DFC pattern and consistent with evidence of altered somatomotor network connectivity and sensory threat monitoring in PTSD (9,15,57). Larger DFC with left lingual gyrus (peripheral visual network) extends the pattern of visuospatial involvement observed across seeds (Harnett et al., 2025; Morey, et al., 2020). Positive associations with the total symptom severity and the four symptom clusters (B, C, D, and E) for these connections suggest that right hippocampal coupling variability with prefrontal, sensorimotor, attentional, and visual systems relates to the full breadth of PTSD symptomatology rather than any specific symptom domain.

## Sex Differences in Functional Connectivity

Sex differences are among the most robust features of PTSD (52). Women are more likely than men to develop the disorder, an excess only partly explained by exposure type (68) and substantially mediated by risk factors more prevalent in women, including acute stress disorder, neuroticism, sexual assault history, anxiety sensitivity, and pre-trauma anxiety (69). Sex modulations on PTSD-related brain features are also widely documented, from an overactive amygdala and altered salience-hub connectivity in women (70) to sex-specific case-control connectivity patterns along amygdala- and hippocampus-precuneus pathways (71). Sex-moderated effects extend to task-based paradigms as well, with our recent work reporting PTSD severity-by-sex interactions in ventromedial prefrontal and cerebellar activation during fear acquisition (72). In the present study, 18 of the 22 connections showing case-control differences in amygdala and hippocampus DFC exhibited a significant diagnosis-by-sex interaction, with post hoc tests localizing the effect to men. Given the large female subsample (n = 1,309, including 529 with PTSD), this pattern is unlikely to reflect limited statistical power. A potential explanation is that amygdala and hippocampus DFC is not the measure most sensitive to PTSD-related alterations in women, which may be better indexed by activation, static connectivity, or structural measures than by temporal variability in coupling with these seeds (70). Unmodeled sources of within-group variance, including hormonal status and cycle phase, may further attenuate detectable effects in women, and resolving these alterations will require broader biopsychosocial models (69,73).

## Strengths and Limitations

This study has several notable strengths that advance PTSD neuroimaging research. First, it represents the largest investigation of dynamic functional connectivity in PTSD to date. Our sample spans diverse age, sex, race, and index traumas, providing substantially greater generalizability than prior studies, which were frequently underpowered to detect the small-to-moderate effect sizes characteristic of PTSD neuroimaging studies. Second, our findings directly address longstanding inconsistencies regarding amygdala and hippocampal dynamic functional connectivity in PTSD. These inconsistencies likely stem from insufficient statistical power, heterogeneous methods, and cohort variability (30). Our study takes advantage of standardized preprocessing across all cohorts using a single centralized pipeline, with dramatically greater statistical power achieved via multi-site aggregation. This combination of reduced noise from the application of a standardized processing pipeline and enhanced statistical power from a large sample provides robust, generalizable evidence for altered dynamic functional connectivity in PTSD. It moves closer to settling prior inconsistent findings and offers a roadmap for large-scale cross-institutional collaboration that can advance rigor and reproducibility in scientific discovery and elevate the clinical impact of neuroimaging research in psychiatry (74).

Several limitations should be considered when interpreting our findings. First, while standardized preprocessing and linear mixed-effects models addressed site-related variance, advanced harmonization methods such as ComBat and ComBat-GAM (30) could better account for site effects but were unfeasible because of systematic missing data patterns across cohorts (75). Notably, certain brain regions such as ventral prefrontal cortex, ventral temporal regions, and superior cortical areas near the vertex, were consistently absent in specific sites due to susceptibility artifacts at air-tissue interfaces and field-of-view limitations inherent to some acquisition protocols. Such missingness cannot be adequately remedied by conventional imputation methods. Second, several important clinical variables were not available consistently at all sites, including medication status, illness duration, trauma characteristics, magnitude of trauma exposure in the control group, and psychiatric comorbidities, which prevented systematic analysis of their influence on connectivity patterns. Third, our seed-based approach treated the amygdala and hippocampus as unitary structures, although both are composed of functionally distinct subregions (9,76) with distinct connectivity. Finally, the cross-sectional design of this study precludes causal inference regarding whether the observed alterations precede PTSD onset and confer risk, or emerge as a consequence of the disorder. Longitudinal investigations from pre-trauma through PTSD onset and treatment response will be essential for establishing the temporal dynamics of amygdala and hippocampus functional connectivity in PTSD (19).

## Conclusion

PTSD is associated with widespread greater temporal variability of amygdala and hippocampus resting-state connectivity spanning control, salience/ventral attention, dorsal attention, default mode, somatomotor, and visual networks. Whether this pattern reflects unresolved dysregulation in PTSD or successful stabilization in trauma-exposed controls remains open, and resolving it will require designs that link connectivity dynamics to symptom course. Sex moderation of these connectivity alterations localized to males, highlighting the neurobiological heterogeneity of PTSD and the need for sex-stratified approaches in future research.

## Supplementary Files

This is a list of supplementary files associated with this preprint. Click to download.

- Table1.docx
- Table2.docx
- Table3.docx
- Supplementaryv4.docx

Table 1 to 3 are available in the Supplementary Files section.

## Figures and Tables

**Figure 1.**
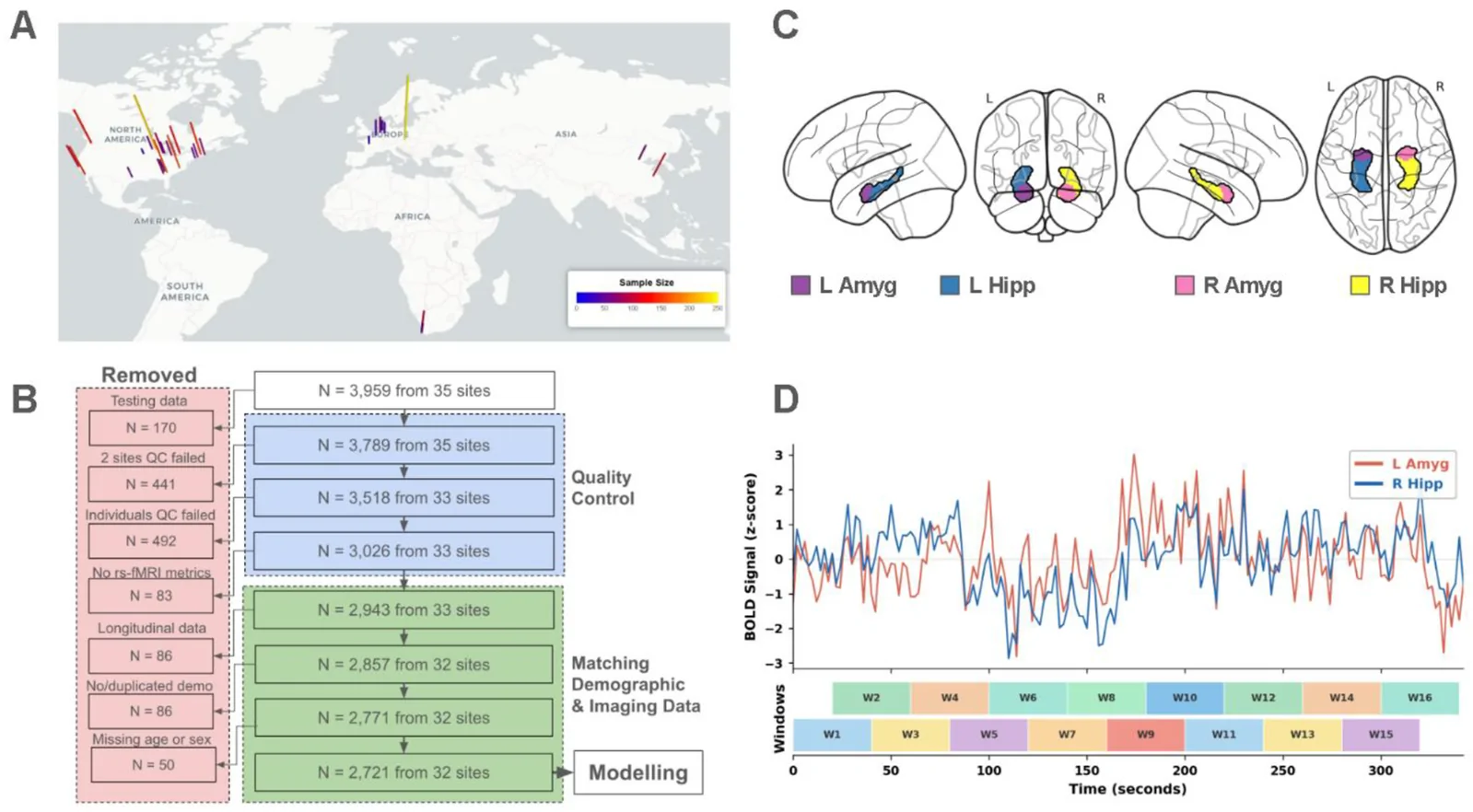
Data aggregation, processing, and analysis framework. (A) Clinical and neuroimaging data were aggregated by the ENIGMA PTSD Working Group through international collaboration of existing datasets. Bar locations represent the geographic locations of study sites, while bar height and color represent the number of participants contributed by each site. For multi-center datasets, the mapped location denotes the coordinating center for data processing and sharing, which may differ from the scanning location. (B) A standardized pipeline was implemented to exclude imaging data from sites and participants that failed quality control assessments, did not yield valid neuroimaging metrics, or lacked complete demographic information. (C) Bilateral amygdala and hippocampus seed regions displayed in glass brain projections (left lateral, posterior, right lateral, and superior views). (D) Z-scored blood-oxygen-level-dependent (BOLD) time series of the left amygdala (red) and right hippocampus (blue) from a single participant as an example. The sliding window schematic beneath the time series illustrates the dynamic functional connectivity analysis approach, using a window length of 40 seconds with 50% overlap between consecutive windows. L = left; R = right; Amyg = amygdala; Hipp = hippocampus.

**Figure 2.**
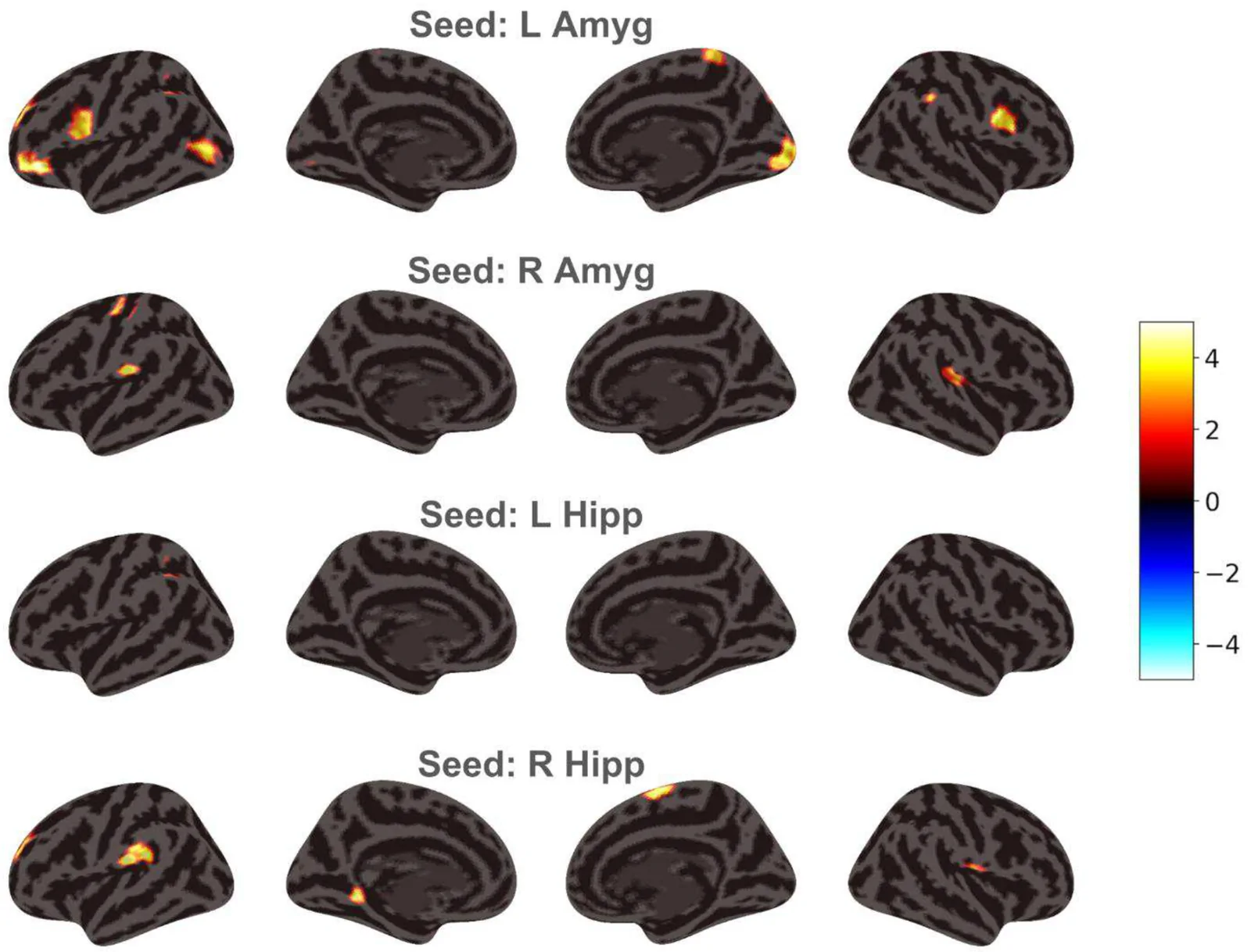
Effects of PTSD diagnosis on DFC. Individuals with PTSD compared to controls demonstrated increased dynamic functional connectivity (DFC; 40-second sliding window, 50% overlap) across multiple connections spanning control, salience/ventral attention, dorsal attention, somatomotor, default mode, and visual networks. For each analysis, connections were considered significant if they survived FDR correction (*q* < .05, two-tailed) applied across all seed-to-region and seed-to-seed connections. Color scale represents *t*-statistics; warmer colors indicate greater DFC in PTSD relative to controls. L = left; R = right; Amyg = amygdala; Hipp = hippocampus.

**Figure 3.**
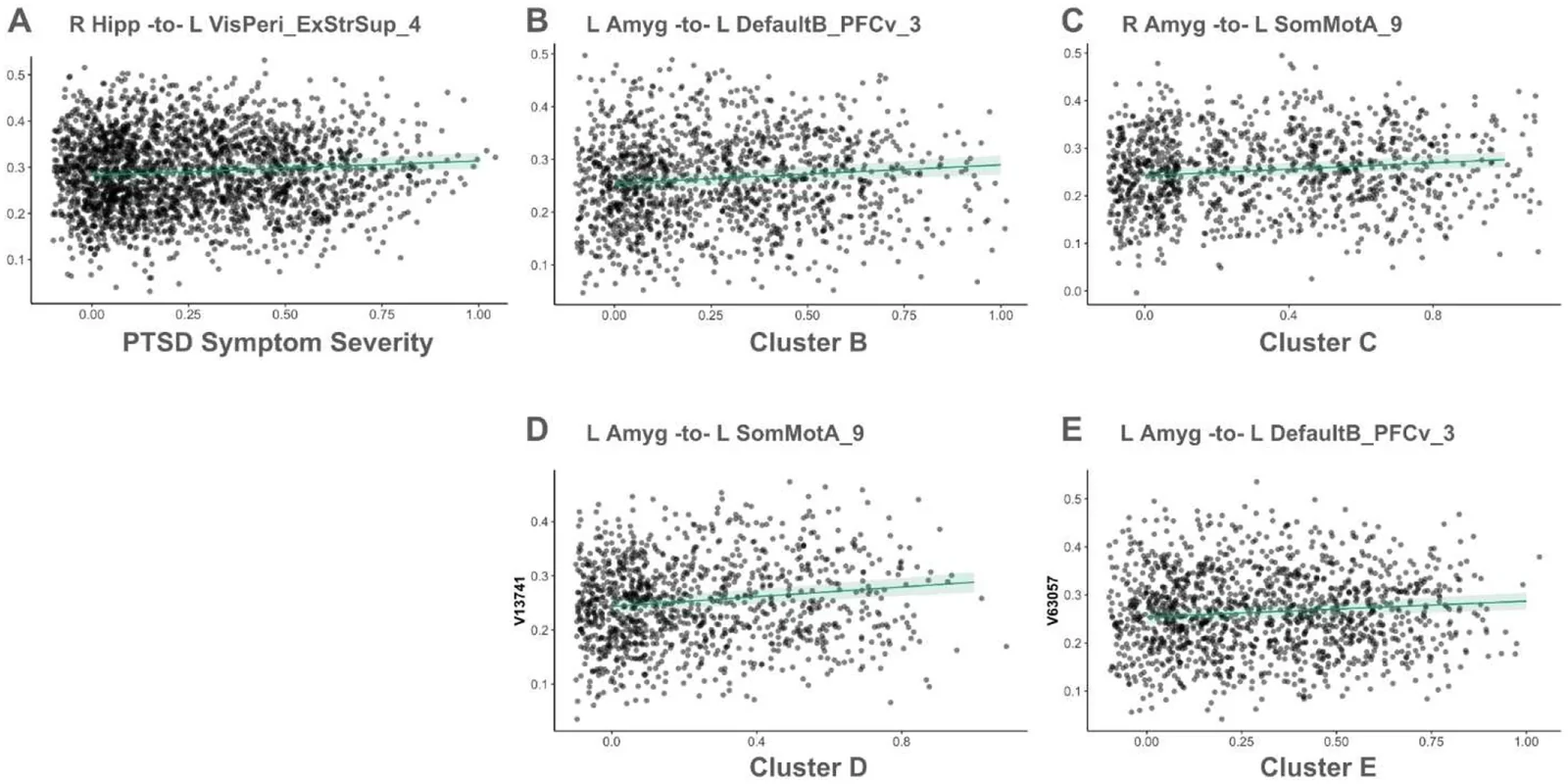
PTSD symptom severity and clusters B-E are positively associated with DFC. Representative plots display model-predicted values of connectivity as a function of (A) PTSD overall symptom severity and (B-E) clusters B-E, with individual participant data points overlaid. Shaded bands represent 95% confidence intervals. L = left; R = right; Amyg = amygdala; Hipp = hippocampus.

## Data Availability

Data is available upon request from the ENIGMA PTSD Working Group. The scripts for data analyses could be obtained from (https://github.com/sundelinustc/Dynamic_Static_rsFC.

## ENIGMA PTSD Working Group Author List & Affiliations:

Delin Sun^1,2,3^, Ahmed Hussain^2,3^, Nick Steele^2,3^, Courtney C. Russell^2,3^, C. Lexi Baird^2,3^, Jenna A. Beakas^2,3^, Leonel Rangel-Jimenez^2,3^, LeeAnne Tunstall^2,3^, Alexandra K. Dwulit^2,3^, Elena Pozzi^4,5^, Hadis Jameei^4,5^, Yuanzhe Liu^4,5^, Lea Waller^6^, Emily L. Dennis^7^, Neda Jahanshad^8^, Sophia I. Thomopoulos^8^, Lauren E. Salminen^8^, Miranda Olff^9,10^, Jessie L. Frijling^11,9^, Dick J. Veltman^9,12^, Saskia B.J. Koch^9,13^, Laura Nawijn^9^, Mirjam van Zuiden^9,14^, Li Wang^15,16^, Ye Zhu^15,16^, Gen Li^15,16^, Jonathan Ipser^17^, Dan J. Stein^17^, Heather Zar^17^, Yuval Neria^18,19^, Xi Zhu^20^, Ashley A. Huggins^21^, Sanne J.H. van Rooij^22^, Negar Fani^22^, Tanja Jovanovic^23,22^, Kerry Ressler^24,25^, Sven C. Mueller^26^, Anna R. Hudson^26^, Judith K. Daniels^27,28^, Anika Sierk^29^, Antje Manthey^29^, Henrik Walter^29^, Nic J.A. van der Wee^30,31^, Steven J.A. van der Werff^30,31^, Robert R.J.M. Vermeiren^32^, Christian Schmahl^33,34^, Julia I. Herzog^33,34^, Pavel Říha^35,36^, Ivan Rektor^35,36^, Milissa L. Kaufman^25,37^, Lauren A. M. Lebois^25,24^, Justin T. Baker^25,38^, Isabelle M. Rosso^25,39^, Elizabeth A. Olson^25,39^, Anthony P. King^40^, Israel Liberzon^41^, Mike Angstadt^40^, Nicholas D. Davenport^42,43^, Seth G. Disner^42,43^, Scott R. Sponheim^42,43^, Thomas Straube^44^, David Hofmann^44^, Guangming Lu^45^, Rongfeng Qi^46^, Xin Wang^47^, Austin Kunch^47^, Hong Xie^47^, Yann Quidé^48,49^, Wissam El-Hage^50^, Shmuel Lissek^51^, Hannah Berg^51^, Steven E. Bruce^52^, Josh Cisler^53^, Marisa C. Ross^54^, Ryan J. Herringa^55^, Daniel W. Grupe^56^, Jack B. Nitschke^57^, Richard J. Davidson^56,57,58^, Christine L. Larson^59^, Terri A. deRoon-Cassini^60,61^, Carissa W. Tomas^62,61^, Jacklynn M. Fitzgerald^63^, Jeremy Elman^64,65^, Matthew Panizzon^64^, Christine Fennema-Notestine^64^, Carol E. Franz^66,67^, Michael J. Lyons^68^, William S. Kremen^66,67^, Brandee Feola^69^, Jennifer U. Blackford^70^, Bunmi O. Olatunji^71^, Geoffrey May^72,73,74^, Steven M. Nelson^74,73,72,75^, Evan M. Gordon^76^, Chadi G. Abdallah^77,78^, Ifat Levy^77,78^, Ilan Harpaz-Rotem^77,78^, Maria Densmore^79^, Benjamin Suarez-Jimenez^80^, Ruth Lanius^81,79^, Jean Théberge^79^, Richard W.J. Neufeld^79^, Raluca M. Simons^82^, Vincent A. Magnotta^82^, Kelene A. Fercho^83^, Lee A. Baugh^82,7^, Matthew Peverill^57^, Kelly Sambrook^84^, Katie A. McLaughlin^25^, Paul M. Thompson^8^, Elisabeth A. Wilde^7^, Lianne Schmaal^4,5^, Ilya Veer^85^, Jennifer S. Stevens^22^, Rajendra A. Morey^1,2,3^

^1^ Department of Psychiatry and Behavioral Sciences, School of Medicine, Duke University, Durham, NC, USA

^2^ Brain Imaging and Analysis Center, Duke University, Durham, NC, USA

^3^ Department of Veteran Affairs Mid-Atlantic Mental Illness Research, Education and Clinical Center, Durham, NC, USA

^4^ Centre for Youth Mental Health, The University of Melbourne, Parkville, Australia

^5^ Orygen, The National Centre for Excellence in Youth Mental Health, Parkville, Australia

^6^ Charité Universitätsmedizin Berlin, corporate member of Freie Universität Berlin and Humboldt-Universität zu Berlin, Department of Psychiatry and Neurosciences CCM, Berlin, Germany

^7^ Department of Neurology, University of Utah, Salt Lake City, UT, USA

^8^ Imaging Genetics Center, Mark and Mary Stevens Neuroimaging & Informatics Institute, Keck School of Medicine of USC, Marina del Rey, CA, USA

^9^ Amsterdam UMC University of Amsterdam, Psychiatry, Amsterdam Neuroscience, Amsterdam, The Netherlands

^10^ ARQ National Psychotrauma Centre, Diemen, The Netherlands

^11^ De Viersprong mental health specialist in personality disorders, family and behavior, Amsterdam, The Netherlands

^12^ Amsterdam UMC Vrije Universiteit, Psychiatry, Amsterdam Neuroscience, Amsterdam, The Netherlands

^13^ Donders Institute for Brain, Cognition and Behavior, Centre for Cognitive Neuroimaging, Radboud University Nijmegen, Nijmegen, The Netherlands

^14^ Department of Clinical Psychology, Utrecht University, Utrecht, The Netherlands

^15^ Laboratory for Traumatic Stress Studies, Chinese Academy of Sciences Key Laboratory of Mental Health, Institute of Psychology, Chinese Academy of Sciences, Beijing, China

^16^ Department of Psychology, University of Chinese Academy of Sciences, Beijing, China

^17^ SA MRC Unit on Risk & Resilience in Mental Disorders, Department of Psychiatry and Neuroscience Institute, University of Cape Town, Cape Town, South Africa

^18^ Department of Psychiatry, Columbia University Medical Center, New York, NY, USA

^19^ New York State Psychiatric Institute, New York, NY, USA

^20^ Department of Bioengineering, University of Texas at Arlington, TX, USA

^21^ Department of Psychology, University of Arizona, Tucson, AZ, USA

^22^ Department of Psychiatry and Behavioral Sciences, Emory University School of Medicine, Atlanta, GA, USA

^23^ Department of Psychiatry and Behavioral Neuroscience, Wayne State University School of Medicine, Detroit, MI, USA

^24^ Division of Depression and Anxiety Disorders, McLean Hospital, Belmont, MA, USA

^25^ Department of Psychiatry, Harvard Medical School, Boston, MA, USA

^26^ Department of Experimental Clinical and Health Psychology, Ghent University, Ghent, Belgium

^27^ Department of Clinical Psychology, University of Groningen, Groningen, The Netherlands

^28^ Traumacentrum Beilen, GGZ Drenthe, The Netherlands

^29^ University Medical Centre Charité, Berlin, Germany

^30^ Department of Psychiatry, Leiden University Medical Center, Leiden, The Netherlands

^31^ Leiden Institute for Brain and Cognition, Leiden, The Netherlands

^32^ Department of Child and Adolescent Psychiatry, Leiden University Medical Center, Leiden, The Netherlands

^33^ Department of Psychosomatic Medicine and Psychotherapy, Central Institute of Mental Health, Medical Faculty Mannheim, Heidelberg University, Germany

^34^ German Center for Mental Health, partner site Mannheim-Heidelberg-Ulm, Germany

^35^ First Department of Neurology, St. Anne’s University Hospital and Faculty of Medicine, Masaryk University, Brno, Czech Republic

^36^ CEITEC-Central European Institute of Technology, Multimodal and Functional Neuroimaging Research Group, Masaryk University, Brno, Czech Republic

^37^ Division of Women’s Mental Health, McLean Hospital, Belmont, MA, USA

^38^ Institute for Technology in Psychiatry, McLean Hospital, Belmont, MA, USA

^39^ Center for Depression, Anxiety, and Stress Research, McLean Hospital, Harvard University, Belmont, MA, USA

^40^ Department of Psychiatry, University of Michigan, Ann Arbor, MI, USA

^41^ Department of Psychiatry, Texas A&M University, Bryan, TX, USA

^42^ Minneapolis VA Health Care System, Minneapolis, MN, USA

^43^ Department of Psychiatry, University of Minnesota, Minneapolis, MN, USA

^44^ Institute of Medical Psychology and Systems Neuroscience, University of Münster, Münster, Germany

^45^ Department of Medical Imaging, Jinling Hospital, Medical School of Nanjing University, Nanjing, China

^46^ Nanjing Drum Tower Hospital, Affiliated Hospital of Medical School, Nanjing University, Nanjing, China

^47^ Department of Neurosciences and Psychiatry, University of Toledo, Toledo, OH, USA

^48^ NeuroRecovery Research Hub, School of Psychology, The University of New South Wales (UNSW) Sydney, Sydney, NSW, Australia

^49^ Centre for Pain IMPACT, Neuroscience Research Australia, Randwick, NSW, Australia

^50^ Université de Tours, INSERM, Imaging Brain & Neuropsychiatry iBraiN U1253, 37032, Tours, France

^51^ Department of Psychology, University of Minnesota, Minneapolis, MN, USA

^52^ Colorado State University, Department of Psychology, Fort Collins, CO, USA

^53^ Department of Psychiatry, University of Texas at Austin, Austin, TX, USA

^54^ Northwestern Neighborhood and Network Initiative, Northwestern University Institute for Policy Research, Evanston, IL, USA

^55^ School of Medicine and Public Health, University of Wisconsin Madison, Madison, WI, USA

^56^ Center for Healthy Minds, University of Wisconsin-Madison, Madison, WI, USA

^57^ Department of Psychiatry, University of Wisconsin-Madison, Madison, WI, USA

^58^ Department of Psychology, University of Wisconsin-Madison, Madison, WI, USA

^59^ Department of Psychology, University of Wisconsin- Milwaukee, Milwaukee, WI, USA

^60^ Division of Trauma and Acute Care Surgery, Department of Surgery, Medical College of Wisconsin, WI, USA

^61^ Comprehensive Injury Center, Medical College of Wisconsin, WI, USA

^62^ Institute of Health and Humanity, Medical College of Wisconsin, WI, USA

^63^ Department of Psychology, Marquette University, Milwaukee, WI, USA

^64^ Department of Psychiatry, University of California San Diego, San Diego, CA, USA

^65^ Stein Institute for Research on Aging & Center for Healthy Aging, University of California San Diego, San Diego, CA, USA

^66^ Department of Psychiatry, University of California, San Diego, La Jolla, CA, USA

^67^ Center for Behavior Genetics of Aging, University of California, San Diego, La Jolla, CA, USA

^68^ Department of Psychological & Brain Sciences, Boston University, Boston, MA, USA

^69^ Department of Psychiatry and Behavioral Sciences, Vanderbilt University Medical Center, TN, USA

^70^ University of Nebraska Medical Center, Munroe-Meyer Institute, Omaha, NE, USA

^71^ Department of Psychology, Vanderbilt University, TN, USA

^72^ Center for Vital Longevity, School of Behavioral and Brain Sciences, University of Texas at Dallas, Dallas, TX, USA

^73^ Department of Psychology and Neuroscience, Baylor University, Waco, TX, USA

^74^ Veterans Integrated Service Network-17 Center of Excellence for Research on Returning War Veterans, Waco, TX, USA

^75^ Department of Psychiatry and Behavioral Science, Texas A&M University Health Science Center, Bryan, TX, USA

^76^ Department of Radiology, Washington University School of Medicine, St. Louis, MO, USA

^77^ Department of Psychiatry, Baylor College of Medicine, Houston, TX, USA

^78^ Department of Psychiatry, Yale University School of Medicine, New Haven, CT, USA

^79^ Department of Psychiatry, Western University, London, ON, Canada

^80^ Department of Neuroscience, University of Rochester Medical Center, Rochester, NY, USA

^81^ Department of Neuroscience, Western University, London, ON, Canada

^82^ Department of Biomedical Sciences, University of South Dakota, Vermillion, SD, USA

^83^ Federal Aviation Administration, Oklahoma City, OK, USA

^84^ Department of Radiology, University of Washington, Seattle, WA, USA

^85^ Department of Developmental Psychology, University of Amsterdam, Amsterdam, The Netherlands
